# Clinicopathological Features of Low-Grade Appendiceal Mucinous Neoplasms Confined to the Appendix

**DOI:** 10.3389/fonc.2021.696846

**Published:** 2021-07-08

**Authors:** Yiyan Lu, Fang Li, Ruiqing Ma, Lan Fang, Changhai Qi

**Affiliations:** ^1^ Department of Pathology, Aerospace Center Hospital, Beijing, China; ^2^ Department of Myxoma, Aerospace Center Hospital, Beijing, China

**Keywords:** low-grade appendiceal mucinous neoplasm, stage, treatment, prognosis, appendix

## Abstract

**Objective:**

To investigate the clinicopathological features and follow-up of low-grade appendiceal mucinous neoplasms (LAMNs) confined to the appendix.

**Methods:**

The clinicopathological features, pathological primary tumor (pT) staging and follow-up of 22 patients with LAMNs confined to the appendix were analyzed retrospectively.

**Results:**

Of 22 patients with LAMNs, 14 were pTis (eight pTis^m^ and six pTis^f^), six were pT3, and two were pT4a. The appendiceal diameter was significantly larger for pTis^f^ than for pTis^m^. The interval between first symptoms and surgery was longer for pTis^f^ than for pTis^m^, but not significantly different. No significant differences were found between the pT stages and appendiceal diameter or in the interval between the first symptoms and surgery. Pathomorphologically, the epithelial structures were mainly flat (100%), undulating or scalloped (82%); a few showed filiform villous hyperplasia (46%), and seven (32%) had serrated lesions in the background. Diverticula may be associated with LAMNs, and the location of acellular mucin caused by diverticula affected the pT stage of the LAMNs. The immunohistochemistry information showed the same pattern with cytokeratin 7 (CK7) negative, cytokeratin 20 (CK20) positive and caudal type homeobox 2 (CDX-2) positive. No lymph node metastasis was found. The lack of treatment guidelines for LAMNs confined to the appendix and different acceptances of patients of preventive intervention led to varied clinical treatments. However, we found no short-term benefits of prophylactic extended resection or hyperthermic intraperitoneal chemotherapy.

**Conclusion:**

LAMNs confined to the appendix are rare and must be differentiated from serrated lesions and diverticula. LAMNs with different pT stages have inert biological behavior. Determining the long-term effects of preventive treatment on survival and recurrence requires more data and a longer follow-up.

## Introduction

Previous studies have used varied diagnostic terminology for appendiceal mucinous neoplasia (AMN) and pseudomyxoma peritonei (PMP) (1–3). The 2010 World Health Organization (WHO) (4) and 2016 Peritoneal Surface Oncology Group International guidelines (5) defined low-grade appendiceal mucinous neoplasms (LAMNs) and named high-grade appendiceal mucinous neoplasms (HAMNs) as independent diseases. Their definition and updates on appendiceal mucinous epithelia are regarded as a milestone. The 2019 WHO classification of tumors of the digestive system (6) outlines the latest classification and pTNM staging of the appendix.

Our center is a single-center research institution for PMP in China, with 1,200 PMP patients having been treated from 2008 to 2021. In 2013, our unit established the Department of Myxoma to manage PMP patients. Most patients exhibited progressive disease accompanied by PMP, with a damaged appendiceal structure. Early stage cases confined to the appendix were rare. This article discusses the clinicopathology, pT staging, and follow-up of 22 patients with LAMNs confined to the appendix in a single center at the Aerospace Center Hospital.

## Materials and Methods

### Ethics Approval

The Ethics Committee of the Aerospace Center Hospital, Beijing, China (No. 20190301-YN-16) approved this study.

### Patient Population

We retrospectively analyzed 368 patients with appendix-recognizable AMN between January 2014 and January 2021. The pathological sections were reviewed by two experienced pathologists. According to the 2019 WHO standards, the LAMN features were mucinous neoplasms with low-grade cytologic atypia, nuclei at the base, and mucus-rich cytoplasm. LAMNs confined to the appendix were included. The exclusion criteria were (1) other appendiceal tumors, such as HAMNs, mucinous adenocarcinomas, and signet ring-cell carcinomas; (2) LAMNs accompanied with PMP; and (3) incomplete medical records. Twenty-two patients with LAMNs without PMP were included, and their medical records, clinicopathological data, and follow-up data were collected.

### Study Parameters

The analysis included the following clinicopathological parameters: age, sex, admitting diagnosis, treatment department, interval between first symptoms and surgery, intraoperative frozen-section diagnosis, resection margins, prophylactic extended resection, resection extent, intraoperative and postoperative hyperthermic intraperitoneal chemotherapy (HIPEC), appendix diameter, LAMNs accompanied by acute inflammation, mucosal epithelial structure, serrated lesions in the background, degree of fibrosis, calcification and pT stage, immunohistochemistry information of CK7, CK20, and CDX-2, lymph node metastasis.

Among these characteristics, pT stage was determined according to the American Joint Committee on Cancer (AJCC) Staging Standard (8th edition) (7) as follows: pTis: LAMNs confined to the appendix (defined as involvement by acellular mucin or mucinous epithelium that may extend into the muscularis propria); pT3: tumor penetrating the muscularis propria or fibrotic appendix wall and invading the subserosa or mesoappendix; pT4a: tumor perforating the visceral peritoneum, including mucinous peritoneal tumors or acellular mucinous tumors on the serosa of the appendix or mesoappendix; and pT4b: tumor directly invading other organs or structures. In our study, according to the degree of fibrosis of the appendiceal wall, pTis was stratified as pTis^m^: fibrosis confined to the mucosa, acellular mucinous/mucinous epithelium in the inner side of the fibrotic mucosa, and pTis^f^: fibrosis extending beyond the mucosa, involving the muscularis propria or the whole layer, acellular mucinous/mucinous epithelium located in the inner side of the fibrotic wall. Overall survival was calculated from the date of surgery to the time of death or the last follow-up.

### Statistical Analysis

Statistical analyses were conducted using SPSS 24.0 (IBM Corporation, Armonk, NY, USA). Continuous data are presented as medians and ranges. Categorical data are presented as numbers and percentages. The Shapiro–Wilk normality test showed that the appendiceal diameter and the interval between first symptoms and surgery were not normally distributed (*P* < 0.05). Correlations between groups of categorical data were analyzed using one-way analysis of variance (ANOVA). In homogeneity tests of variance, *P >*0.05 was considered consistent with the hypothesis of homogeneity of the variance test. The corrected Welch one-way ANOVA was used when the variance was missing. *P <*0.05 was considered statistically significant.

## Results

### Clinical Features

**Table 1** presents the demographic and clinical characteristics of the 22 included patients. The ratio of men to women was 1:1.75. The median patient age at LAMNs diagnosis was 61.3 years (range, 33–90 years). Ten patients were diagnosed with appendicitis on admission (three in the myxoma department, seven in other departments); seven were diagnosed with appendiceal cysts (five in the myxoma department, two in other departments); one was diagnosed with intestinal obstruction in the myxoma department; the other four were treated in other departments because they had other tumors (two with ascending colon cancer; one with ovarian cysts, and one with gastric cancer). Four cases of LAMNs were found accidentally, and the interval between first symptoms and surgery was 1–1,825 days (mean: 172 days) in the remaining 18 cases.

**Table 1:** Clinicopathological features of LAMNs confined to appendix.

Characteristics	No. of patients
Sex	
Female	14 (64%)
Male	8 (36%)
Median age (range)/Years	61.3 (33–90)
Admitting diagnosis	
Appendicitis	10 (45%)
Appendiceal cysts	7 (32%)
Intestinal obstruction	1 (5%)
Other tumor	4 (18%)
Median of interval between first symptom and surgery (range)/Day	172 (1-1825)
Intraoperative frozen-section diagnosis	12 (55%)
Resection margin	
Positive	3 (14%)
Negative	19 (86%)
Extended resection	7 (32%)
Intraoperative and postoperative HIPEC	6 (27%)
Median of diameter(range)/CM	2.5 (0.8–6)
Accompanied by acute inflammation	5 (23%)
Epithelial structures	
Flat	22 (100%)
Undulating or scalloped	18 (82%)
Filiform villous	10 (46%)
Serrated lesions in background	7 (32%)
Degree of fibrosis	
Confined to the mucosa	10 (45%)
Full-thickness	12 (55%)
Accompanied with calcification	12 (55%)
pT stage	
pTis	14 (64%)
pTis^m^	8 (57%)
pTis^f^	6 (43%)
pT3	6 (27%)
pT4a	2 (9%)
pT4b	0 (0%)
Follow-up	
Lost	1 (5%)
Death	1 (5%)
Disease-free survival	20 (90%)
Median of Disease-free survival (range) /Month	28 (3–80)

### Pathological Features

**Figure 1:**
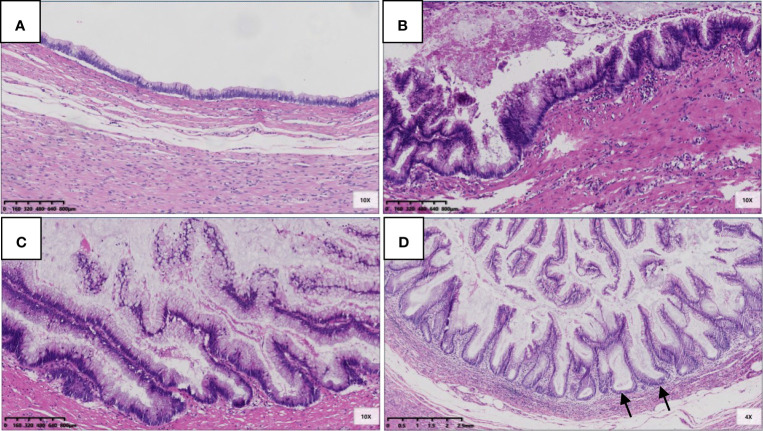
Epithelial structural changes in LAMN. **(A)** The flat low-grade mucinous epithelium was directly attached to the inner side of the fibrotic mucosa, and the muscularis propria of the appendix was intact (HE 100×); **(B)** Undulating or scalloped structures can be found (HE 100×); **(C)** Filiform villous structures can be found (HE 100×); **(D)** Serration was found in the background, which had typical crypt architectural features, including serration, dilatation, horizontal orientation, L-shape or inverted T-form (black arrow) (HE 100×).

For the 22 LAMNs, the average appendiceal diameter was 2.5 cm (range: 0.8–6.0 cm), and five had acute inflammation. The epithelial structures were flat (22/22, 100%; **Figure 1A**), undulating or scalloped (18/22, 82%; **Figure 1B**), and some were accompanied by filiform villous hyperplasia (10/22, 46%; **Figure 1C**). Seven cases exhibited serrated lesions (7/22, 32%; **Figure 1D**) in the background. All patients had varying degrees of appendiceal wall fibrosis; 10 were confined to the mucosal layer, 11 had mucosal fibrosis with partial full-thickness, and 1 case showed complete full-thickness fibrosis.

**Figure 2:**
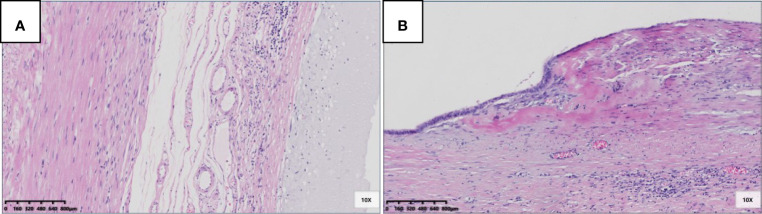
Pathological morphology and stratification of pTis. **(A)** pTis^m^: fibrosis was confined to the mucosa, acellular mucinous/mucinous epithelium located in the inner side of the fibrotic mucosa (HE 100×); **(B)** pTis^f^: fibrosis extends beyond the mucosa, involving the muscularis propria, or even the whole layer, acellular mucinous/mucinous epithelium located in the inner side of the fibrotic wall (HE 100 x).

Fourteen patients had pTis; eight had pTis^m^ (**Figure 2A**), and six had pTis^f^ (**Figure 2B**). The appendiceal diameter was larger in the pTis^f^ group than in the pTis^m^ group (3.1 *vs*. 1.5 cm). In the correlation analysis of pTis stratification (pTis^m^, pTis^f^) and appendiceal diameter, Levene’s test of homogeneity of variance met the hypothesis of homogeneity of variance (*P* = 0.410). The one-way ANOVA results showed statistically significant differences between pTis stratification and appendiceal diameter; F (1,12) = 5.943, *P* = 0.031 (*P* < 0.05). The interval between the first symptom and surgery was longer in the pTis^f^ group than in the pTis^m^ group (means: 89 *vs*. 369 days); Levene’s test of homogeneity of variance was *P*=0.047; the corrected Welch one-way ANOVA showed no significant differences between pTis stratification and the interval between the first symptom and surgery; Welch F (1,5.260) = 0.888, *P* = 0.387 (*P* > 0.05).

**Figure 3:**
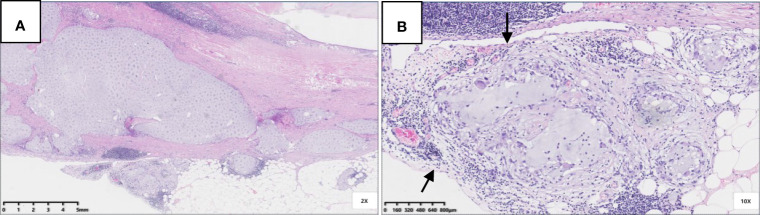
Pathological morphology of pT3. **(A)** pT3: fibrosis of appendix wall, acellular mucinous invaded subserosa or mesoappendix (Hex) 20x); **(B)** A complete serosa layer can be seen (black arrow) (HE 100x).

**Figure 4:**
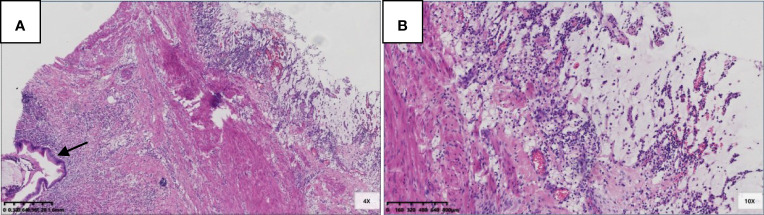
Pathological morphology of pT4a. **(A)** Flat and clustered low-grade mucinous epithelium lined in the inner side of the fibrotic mucosa (black arrow), and acellular mucinous with inflammatory reaction was found (HE 40×); **(B)** The structure of serosa was destroyed and replaced by acellular mucinous with inflammatory reaction (HE 100×).

**Figure 5:**
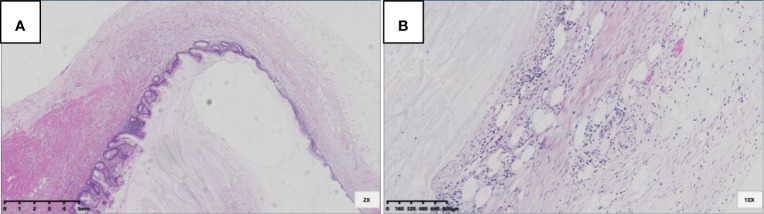
pT3 with diverticulum. **(A)** Rupture of muscularis propria (black arrow), mucous epithelium, and mucus diverticulate along the rupture (blue arrow) (HE 20×); **(B)** In the part of diverticulum, acellular mucinous was directly located in the mesoappendix (HE 100×).

**Figure 6:**
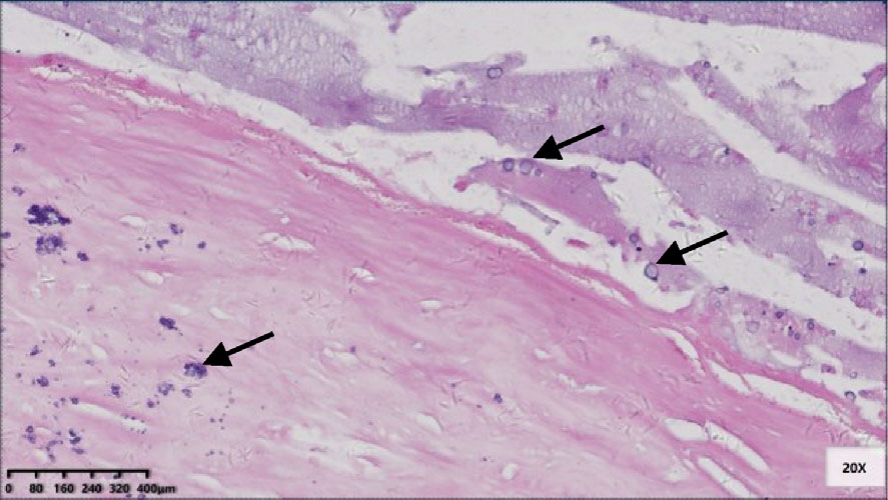
Micro-calcification scattered in the fibrotic appendix wall and in the lumen contents (black arrow) (HE 200×).

Six patients were pT3 (**Figure 3**), of which three had diverticula with the epithelium in the inner side of the diverticular wall, and the diverticulum led to acellular mucinous overflow to the subserosa (**Figure 4**). Two patients were pT4a (**Figure 5**), and one had diverticulum, leading the acellular mucinous overflow to the appendiceal serosa. No patients were pTis4b in our group. Twelve patients (12/22, 55%) had calcification of the appendiceal lumenal contents/wall (**Figure 6**). However, we found no significant difference between the pT stages and interval between the first symptoms and surgery: F(2,19) = 1.431, *P* = 0.264 (P > 0.05).

**Figure 7:**
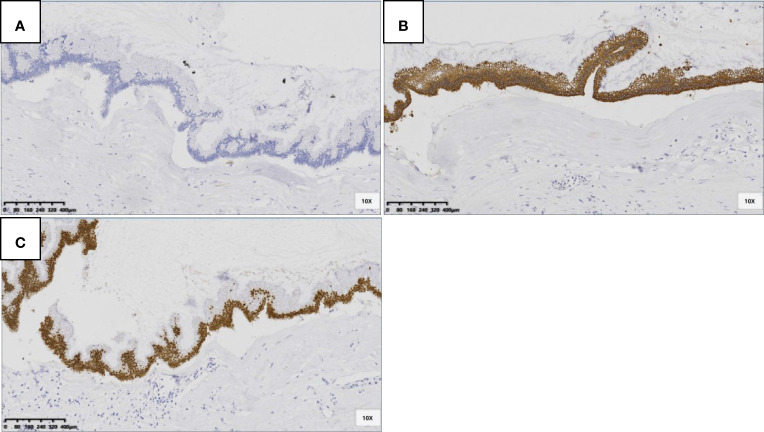
LAMNs showed CK7 negative **(A)**, CK20 positive in cytoplasm **(B)** and CDX-2 positive in nuclear **(C)** (SP 100×).

The immunohistochemistry information showed the same pattern with cytokeratin 7 (CK7) negative, cytokeratin 20 (CK20) positive and caudal type homeobox 2 (CDX-2) positive (**Figure 7**). No lymph node metastasis was found in our cases.

### Clinical Treatment

Intraoperative frozen diagnosis was performed for 12 patients (nine by the myxoma department). LAMNs were definitively diagnosed from the intraoperative frozen sections. Three patients had positive resection margins. Based on the results of the intraoperative frozen diagnostics, family members gave informed consent for the patients to undergo prophylactic extended resection. Among them, seven patients underwent different degrees of prophylactic extended resection (five in the myxoma department). In the myxoma department, six patients underwent intraoperative HIPEC (60 mg cisplatin and 80 ml elemene). Among them, two underwent five courses of postoperative HIPEC (1 g fluorouracil), and the remaining four terminated treatment because of poor water circulation during postoperative HIPEC. The ten cases not submitted for intraoperative frozen diagnosis were all treated in other departments, and no prophylactic extended resection or intraoperative or postoperative HIPEC were performed.

### Follow-Up

Among the 22 patients, one was lost to follow-up, and one died of cardiovascular disease 63 months later; The remaining 20 patients were followed for 3–80 months (mean: 28 months), and two with pT4a were followed for 63 and 75 months. All cases showed disease-free survival.

## Discussion

Our case series showed that LAMNs were more likely to occur in older women, which is consistent with previous reports (8–10). The symptoms of LAMNs were mainly appendicitis and appendiceal cysts, and a few were incidentally found during resection of other tumors. The intervals between the first symptoms and surgery differed. Five patients underwent an appendectomy after 1,825 days (5 years). However, we found no significant difference between the pT stages and interval between the first symptoms and surgery.

The appendiceal diameters were increased to varying degrees, but no significant differences were found between the pT stages and appendiceal diameter. However, the appendix should not be allowed to expand freely because rupture and perforation will occur, and perforation plays a role in peritoneal dissemination (10, 11).

LAMNs are difficult to differentiate from serrated lesions. Importantly, LAMNs have pushing infiltration and different degrees of appendiceal wall fibrosis (12). Serrated lesions have intact mucosal muscle. Some tumors are heterogeneous; some areas are similar to serrated lesions, and others are similar to LAMNs. These tumors should be classified as LAMNs (13). All patients in our group had different degrees of appendiceal fibrosis. Calcification is a good indication for preoperatively diagnosing LAMNs. However, the calcification is not coarse but tends to be microcalcification scattered in the fibrotic appendiceal wall or in the lumen. This subtle change requires careful observation by radiologists.

LAMNs often have different degrees of appendiceal wall fibrosis. Therefore, pT1 and pT2 staging in colorectal TNM staging is unsuitable for LAMNs. pTis (in LAMNs) includes stages pTis, pT1, and pT2 of colorectal cancer. Fourteen patients (64%) had pTis in this group. Because of the different degrees of fibrosis, pTis in LAMNs can be stratified as pTis^m^, in which fibrosis is confined to the mucosa with acellular mucinous/mucinous epithelium in the inner side of the fibrotic mucosa, or pTis^f^, in which fibrosis extends beyond the mucosa, involving the muscularis propria or the whole layer, with acellular mucinous/mucinous epithelium in the inner side of the fibrotic wall. The interval between first symptoms and surgery was longer in the pTis^f^ group (mean: 369 days) than in the pTis^m^ group (mean: 89 days), but the difference was not statistically significant. The appendiceal diameter was significantly larger in the pTis^f^ group (mean: 3.1 cm) than in the pTis^m^ group (mean: 1.5 cm). Morphologically, the pTis^f^ group had a higher risk of progression to perforation. However, such detailed stratification may not be of great practical significance in predicting survival and recurrence. Our follow-up data showed that all cases of pTis achieved disease-free survival except the one who died of cardiovascular disease. Phoenix et al. questioned the practical significance of the pT3 classification. These researchers found that among ten patients with pT3 LAMNs, none resulted in PMP (14). Umetsu et al. (9) and Wong et al. (10) found that pT3 was uncommon in LAMNs, with no recurrence after 45 and 20 months of follow-up, respectively, which was similar to the results of our study. In our pT3 group, except for one patient who was lost to follow-up, five patients achieved disease-free survival (follow-up 4–44 months, average 17 months). pT3 seems to show similar biological behavior to pTis, but more data are needed to verify this. Additionally, we found that three of six patients with pT3 had diverticula, and the acellular mucin of the diverticula was located directly in the subserosa. A similar situation occurred in the pT4a group: one patient had diverticulum, and the acellular mucin was on the serosa. For LAMNs with diverticula, the most important thing is to distinguish them from simple diverticula. Rupture of the diverticulum may cause mucus to accumulate in the subserosa, mesoappendix or serosa, which can be easily confused with LAMNs. The key to distinguishing this is that the diverticulum has residual lamina propria and can be accompanied by Schwann cell proliferation, with a lack of epithelial atypia and extensive appendiceal wall fibrosis (15). Epithelial atypia and appendiceal wall fibrosis occurred in our patients with LAMNs with diverticula. Second, the location of the acellular mucin due to diverticulum affected the LAMN pT stage, but its significance requires further study. Yantiss et al. (16) and Pai et al. (8) described pT4a LAMN recurrence rates of 4 and 7%, respectively. In our group, the two patients with pT4a showed no recurrence. In conclusion, the long-term prognoses for patients with LAMNs at different pT stages require more data and a longer follow-up. The immunohistochemistry information showed the same pattern with CK7 negative, CK20 positive and CDX-2 positive, suggesting that it was of digestive tract epithelial origin. Assessment of overall nodal involvement is not straightforward because right hemicolectomies are often not performed. In our group, only six cases underwent right hemiresection, and no lymph node metastasis was found. In the appendectomy cases, the imaging did not show enlarged lymph nodes.

Treating LAMNs confined to the appendix is controversial, and the surgical strategy is mainly inferred from the treatment mode for colon cancer. However, some researchers believe that preventive extended resection is not beneficial to the survival of patients with non-metastatic LAMNs when the resection edge of the appendectomy is negative (17). Additionally, whether preventive HIPEC can provide long-term survival benefits remains controversial (18). In our group, the differences in treatment strategies among departments and different acceptances of patients of preventive intervention resulted in varied clinical treatment data. However, our study found no short-term benefits of prophylactic extended resection or HIPEC. Owing to the inert biological behaviors of LAMNs, more data and a longer follow-up are needed to determine the long-term effects of preventive treatment on survival and recurrence.

In conclusion, LAMNs confined to the appendix are rare and should be differentiated from serrated lesions and diverticula. pTis has different stratifications, but its practical application value is uncertain. Regardless of pT stage, LAMNs confined to the appendix show inert progression. At present, no clear treatment guidelines exist. The long-term survival benefits for patients at different pT stages and undergoing different preventive interventions should be verified with more data and longer follow-ups.

## Data Availability Statement

The original contributions presented in the study are included in the article/supplementary material. Further inquiries can be directed to the corresponding author.

## Ethics Statement

The studies involving human participants were reviewed and approved by The Ethics committee of the Aerospace Center Hospital, Beijing, China(No. 20190301-YN-16). The patients/participants provided their written informed consent to participate in this study.

## Author Contributions

The author contributions were as follows: YL and CQ conceived and designed the experiments. YL and RM provided study material or patients. FL, LF, and YL collected and assembling data. YL and FL analyzed and interpreted the data. YL and CQ contributed to the draft of the manuscript. YL, FL, RM, and LF revised the manuscript critically for important intellectual content. All authors contributed to the article and approved the submitted version. All authors agreed to be accountable for all aspects of the work inensuring that questions related to the accuracy or integrity of any part of the work are appropriately investigated and resolved.

## Funding

The foundation of the Aerospace Center Hospital (20190301-YN-16).

## Conflict of Interest

The authors declare that the research was conducted in the absence of any commercial or financial relationships that could be construed as a potential conflict of interest.
